# Long-Term Cardiac Sequelae in Patients Referred into a Diagnostic Post-COVID-19 Pathway: The Different Impacts on the Right and Left Ventricles

**DOI:** 10.3390/diagnostics11112059

**Published:** 2021-11-06

**Authors:** Giovanna Pelà, Matteo Goldoni, Chiara Cavalli, Felice Perrino, Sara Tagliaferri, Annalisa Frizzelli, Pier Anselmo Mori, Maria Majori, Marina Aiello, Nicola Sverzellati, Massimo Corradi, Alfredo Chetta

**Affiliations:** 1 Department of Medicine and Surgery, University of Parma, 43100 Parma, Italy; matteo.goldoni@unipr.it (M.G.); chiara.cavalli3@studenti.unipr.it (C.C.); feliceper@gmail.com (F.P.); sara.tagliaferri@unipr.it (S.T.); annalisa.frizzelli@unipr.it (A.F.); marina.aiello@unipr.it (M.A.); nicola.sverzellati@unipr.it (N.S.); massimo.corradi@unipr.it (M.C.); alfredo.chetta@unipr.it (A.C.); 2 Department of General and Specialistic Medicine, University-Hospital of Parma, 43100 Parma, Italy; 3 Respiratory Disease and Lung Function Unit, Cardio-Thoracic and Vascular Department, University-Hospital of Parma, 43100 Parma, Italy; 4 Pulmonology and Endoscopic Unit, Cardio-Thoracic and Vascular Department, University-Hospital of Parma, 43100 Parma, Italy; pmori@ao.pr.it (P.A.M.); mmajori@ao.pr.it (M.M.); 5 Diagnostic Department, Radiological Sciences, University-Hospital of Parma, 43100 Parma, Italy

**Keywords:** SARS-CoV-2, long-COVID-19, cardiac involvement, echocardiography, pulmonary hypertension, biventricular dysfunction

## Abstract

Most patients who had COVID-19 are still symptomatic after many months post infection, but the long-term outcomes are not yet well defined. The aim of our prospective/retrospective study was to define the cardiac sequelae of COVID-19 infection. This monocentric cohort study included 160 consecutive patients who had been discharged from the ward or from the outpatient clinic after a diagnosis of COVID-19 and subsequently referred for a follow-up visit. Clinical features’ data about the acute phase along with information about the follow-up visit, including ECG and Echocardiographic parameters, were recorded. At an average follow-up of 5 months, echocardiography showed morpho-functional characteristics of both right (RV) and left (LV) ventricles, such as RV dilation, increased pressure in the pulmonary circulation, and bi-ventricular systolic–diastolic dysfunction. When examined using multivariate analysis, independent of age, sex, and co-morbidities, RV and LV changes were significantly associated with chest High-Resolution computed tomography score and hemodynamic Instability (HI), and with C-reactive protein, respectively. Our results suggest that COVID-19 may impact RV and LV differently. Notably, the extent of the pneumonia and HI may affect RV, whereas the inflammatory status may influence LV. A long-term follow-up is warranted to refine and customize the most appropriate therapeutic strategies.

## 1. Introduction

In Italy, the SARS-CoV-2 virus caused over 35,000 deaths in the first wave of the outbreak that began in March 2020. In 40% of cases, the cause of death was attributable to cardiac involvement [1,2,3].

Cardiac involvement in the acute phase was mainly evaluated as cardiac troponin elevation >99th percentile, with percentages that varied according to the different clinical records from 8% to 30% of hospitalized patients with even higher values in both critically ill or deceased patients [4,5,6].

Several mechanisms of COVID-19-induced cardiovascular diseases have been proposed: micro- and macro-thromboses secondary to the activation of the hemocoagulation cascade; vasculitis with endothelial damage; microvascular dysfunction and sympathetic activation that can progress to stress cardiomyopathy; a state of hyper-inflammation; direct viral myocardial injury; coronary plaque destabilization with evolution towards type 1 myocardial infarction or a mismatch between oxygen supply and demand; reduction in supply for hypoxemia; and hypotension and increased demand for tachycardia and fever, leading to type 2 myocardial infarction [4,5,6,7,8,9,10,11,12].

Due to the high risk of spreading the infection, few imaging studies of cardiac involvement in the acute phase of SARS-CoV-2 disease exist that demonstrate a specific involvement of the right ventricle [13,14,15]. Szekely Y et al. [15] showed that, in a group of 100 consecutive patients hospitalized for COVID-19 in March 2020 who underwent echocardiographic examination within 24 h of their admission to the hospital, the most frequent manifestation was right ventricular (RV) dilation/dysfunction. At the same time, another study evaluated the prognostic significance of RV longitudinal strain (RVLS) in a group of 120 consecutive patients undergoing bedside echocardiography during hospitalization (median time from admission to echo 7 days) and demonstrated that RVLS is a powerful predictor of higher mortality. These findings suggest that assessment of RV function should be implemented to identify patients at higher risk for poor outcomes [13].

Therefore, a specific impairment of RV may be related to an increased afterload from acute respiratory failure, pulmonary embolism, pulmonary micro-thromboses, and microvascular damage, and, thus, RV impairment finds itself “in the eye of the storm”, as recently defined by some authors [14].

Taken together, these data suggested that SARS-CoV-2 could affect both the left ventricle (LV) and RV, thereby establishing the heterogeneity of patients with COVID-19 from a cardiac point of view.

The long-term outcomes and, in particular, the cardiac sequelae of COVID-19 are not completely known. The evaluation of cardiac involvement in patients with a history of COVID-19 is important not only to clarify the clinical status of these patients, most of whom are still symptomatic, but also to enable the implementation of early preventive interventions.

This prospective/retrospective study focused on a sample of COVID-19 patients referred for standard local hospital protocols after recovery from COVID-19 infection. The aims of this study were to define the structural and functional cardiac characteristics, both in LV and RV, at a mean follow-up of 5 months after infection and to investigate the relationships between the cardiac findings at follow-up and in the acute care clinical setting.

## 2. Materials and Methods

### 2.1. Study Population

This was a monocentric cohort study that included 160 consecutive patients who had been discharged from the ward (*n* = 117) or from the outpatient clinic (*n* = 43) after a diagnosis of COVID-19, and subsequently referred for a follow-up visit at University-Hospital of Parma between May and November 2020. The sample included patients with: (1) complicated acute COVID-19 infection phase; (2) a positive TC score regardless of the presence of symptoms; and (3) the persistence of symptoms some weeks after recovery. In May 2020, a dedicated office was opened at the Hospital of Parma that referred patients for a standard diagnostic pathway after receiving a report from a hospital doctor or a general practitioner.

The study protocol was approved by the Local ethics committee AVEN (RESPCOV-2, Protocol n. 681/2020/SPER/AOUPR, approval date: 8 September 2020). Written, informed consent was obtained from all participants.

Exclusion criteria were two-fold: a history of significant heart or/and respiratory disease and/or an unwillingness to participate or provide informed consent.

Clinical details about hospitalization, i.e., Intensive Care Unit (ICU) stays, days of hospitalization, severity of lung involvement, as defined by a score of chest High-Resolution computed tomography (HRCT) (128-slice scanner, SOMATOM Definition Edge, Siemens Healthineers, Erlangen, Germany), medical histories, comorbidities, symptoms, cardiovascular (CV) complications, treatments, and outcomes were collected.

Respiratory gas exchange and oxygen supplementation during hospitalization were also assessed.

At follow-up, a complete cardiac assessment including clinical evaluation with 12-lead resting Electrocardiogram (ECG) and Conventional and Doppler Tissue Echocardiographic (DTE) examination was performed.

The presence of symptoms at follow-up, specifically dyspnea, asthenia, heartbeat, chest pain, myalgia, paresthesia, weight loss, and alterations in the sleep–wake rhythm, was recorded.

### 2.2. Transthoracic Echocardiography

M-mode, two-dimensional, and Doppler ECHO were performed by an ultrasonography-experienced cardiologist (GP), using a commercially available, multi-hertz sector, 2–4 MHz probe-equipped machine (Vivid S5, General Electric Healthcare, Milwaukee, Wisconsin, GE, USA). LV end-systolic (LVESD) and end-diastolic (LVEDD) diameters, interventricular septal and posterior wall thicknesses, LV mass (LVM), and relative wall thickness (RWT) were calculated as previously described [16,17]. Simpson’s biplane rule-based end-diastolic (LVEDV) and systolic (LVESV) volumes and ejection fraction (EF) were calculated, while Fractional Shortening (FS) was [(LVEDD − LVESD)/LVEDD] × 100. Cardiac output (CO) was derived by the formula: LVEDV-LVESV.

RV dimensions were estimated from the short axis parasternal view in order to extract proximal right ventricular outflow tract (RVOT) as the distance from RV anterior wall to the aortic valve; and, from the apical four-chamber view, we measured the longitudinal axis (RVLd) as the distance between the apex of the RV and the center of the tricuspid ring and the transversal axis (RVTd) as the maximal transversal dimension in the basal one-third of RV inflow, at the end-diastole [16,17].

Mitral and tricuspidal inflow patterns were analyzed from the apical four-chamber view, and E and A waves and their ratio were considered as peak flow velocity (pv) and time velocity integral (tvi) in order to evaluate the conventional LV and RV diastolic function [17,18].

From the same projection, DTE analysis was performed at the lateral site (Lat.) and at the postero-septum (Sept.) of the mitral annulus to assess the myocardial systolic (S) and diastolic (E’, A’) waves of LV, as well as at the lateral site of tricuspidal annulus to evaluate the longitudinal motion of RV. E/E’ was calculated in order to estimate LV and RV filling pressure.

Pulmonary artery pressure (PAP) was estimated directly from the pv of the tricuspid jet (systolic pulmonary artery pressure, SPAP) and by the pulmonary flow acceleration time (AT) to assess pulmonary vascular resistance [15,16].

### 2.3. Statistical Analyses

Quantitative variables are reported as means ± SD or median (IQ range) based on the normality (K-S test). Qualitative variables are reported as absolute and % prevalence.

The determinants of LV and RV structure and function (dependent variables) were initially assessed via univariate analysis (Pearson’s r and its significance, given the absence of outliers influencing the test), and consideration was given not only to the different parameters that defined the severity of the disease (bio-humoral indices as CRP and D-dimer, and the severity of COVID-19 infection, assessed as hospitalization, length of hospitalization, ordinary or ICU admission, HRCT score, CV complications oxygen therapy, and invasive or non-invasive ventilation) but also to patients’ age, sex, systolic BP, body mass index (BMI), smoking or being a former smoker, and comorbidities, such as hypertension, coronary artery disease (CAD), diabetes mellitus, and chronic respiratory diseases (CRDs). This preliminary analysis was effectuated to find potential associations between the different echocardiographic parameters under study and the clinical variables of the patients in addition to possible multicollinearity, among other predictors. Due to the presence of several possible significant determinants, backward stepwise multiple linear models (inclusion criterium *p* < 0.1) were used to identify independent predictors of cardiac parameters assessed with echocardiography and the possible relationship between the heart and COVID-19 disease.

All the models were accurately assessed in order to: (1) verify the normality of residuals and, therefore, exclude non-linear effects and/or the presence of outliers and (2) control the presence of residual collinearity through the use of Variance inflation factors (VIF) < 2. A two-tailed *p*-Value < 0.05 was considered as statistically significant. SPSS v 26 statistical package was used for all analyses (IBM, Armonk, NY, USA).

## 3. Results

### 3.1. Clinical Characteristics

Table 1 shows the main characteristics of the study population enrolled: 64 were female (F) and 96 were male (M) (mean age 60 ± 12 years). The mean BMI was higher than normal (28 ± 6 Kg/m^2^) and 28% of the sample was obese (BMI value > 30 Kg/m^2^). The systolic (130 ± 16.0 mmHg) and diastolic BP (83 ± 9.6 mmHg) were in the normal range as HR (73 ± 13 bpm). The most frequent comorbidities in our population were hypertension (43%), CRDs (21%; chronic obstructive pulmonary disease *n* = 28; pulmonary fibrosis *n* = 2; lung cancer *n* = 1; sarcoidosis *n* = 1; Obstructive Sleep Apnea Syndrome, *n* = 1), diabetes mellitus (14%), and CAD (11%). Eight percent were active smokers and 43% were former smokers.

Twenty-three percent of the participants were on β-blockers, 18% on ACE-inhibitors, 14% on Angiotensin II Receptor Blockers (ARB), and 18% on Aspirin in the period prior to infection.

### 3.2. Retrospective Data

**Figure 1:**
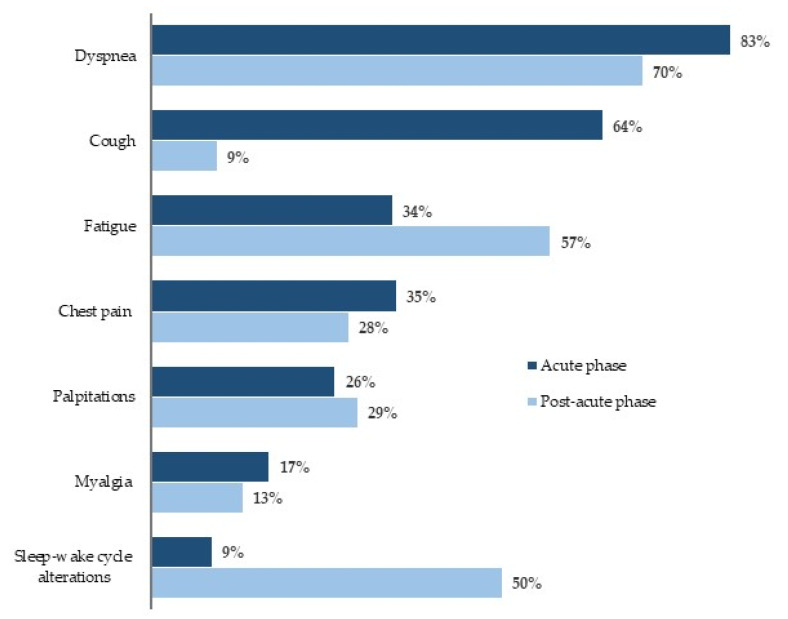
COVID-19-Related symptoms.

The most frequent symptoms in the acute phase of the disease were fever (94%), dyspnea (83%), weight loss (79%), and cough (64%). Other symptoms reported were dysgeusia (55%), anosmia (45%), chest pain (35%), fatigue (34%), diarrhea (26%), palpitations (26%), myalgia (17%), and sleep–wake cycle alterations (9%) (Figure 1).

In all patients, the diagnosis of COVID-19 was confirmed by the positivity of a throat swab for SARS-CoV-2. All hospitalized patients except one (99%), and 24 out of 43 outpatients (56%), underwent HRCT scans to confirm pneumonia; the HRCT score data were available in 119 patients, with an average value of 40 ± 22 (hospitalized patients 44 ± 21; outpatients 17 ± 10).

Data regarding the severity and the course of the disease are shown in Supplementary Table S1. Of 160 patients, 117 (73%) needed hospitalization because their pneumonia required oxygen supplementation, and they had a median length of stay of 20 (IQ 9.0–37.5) days. Fifteen percent of hospitalized patients needed treatment in the ICU. In this group, median length of stay was 45 (37.5–74.5). Oxygen therapy was administered in 89% of hospitalized patients: 66% received oxygen with a mask, while non-invasive ventilation (NIV) with a C-PAP helmet and invasive mechanical ventilation were required in 19% and 14% of the patients, respectively.

Cardiovascular complications were arrhythmia (10%; atrial ectopic beat *n* = 10; ventricular arrhythmias *n* = 2; atrial fibrillation *n* = 4), followed by venous thromboembolism (VTE, 8%), hemodynamic instability (HI, defined mainly by hypotension and also by the need for pharmacological support with sympathomimetic amine, i.e., noradrenaline iv (7%)), deep vein thrombosis (DVT, 2%), and pericarditis (1%).

Regarding laboratory data, CRP and D-dimer were markedly increased, transaminases slightly increased, and creatinine was in the normal range (Supplementary Table S2).

The pharmacological treatment consisted of Antibiotics (90%), Hydroxychloroquine (72%), Colchicine (14%), Antivirals (lopinavir/ritonavir or darunavir/cobicistat) (49%), Biologics (Tocilizumab or Sarilumab, 10%), Systemic corticosteroids (41%), Low-Molecular-Weight Heparin (LMWH) in a prophylactic dose (52%), and LMWH in an anticoagulant dose (19%) (Supplementary Table S3).

### 3.3. Prospective Data

Out of 160 patients, 150 (94%) were still symptomatic with at least one symptom after a median follow-up period of 20 weeks (150 ± 57 days; 5 months), with dyspnea and fatigue being the most frequent symptoms. Figure 1 shows the symptoms at follow-up compared to those in the acute phase: Dyspnea, albeit with reduced percentages, was the most frequent symptom, followed by asthenia, which, instead, was reported to a greater extent at follow-up. Chest pain and palpitations persisted with similar percentages, but a significant increase in alterations in the sleep–wake rhythm was observed.

Electrocardiographic data showed repolarization abnormalities such as ST depression in 24% patients, right intraventricular conduction disturbances in 8%, and 6% showed arrhythmias at follow-up. PR, QRS and QTc intervals were in the normal range (Supplementary Table S4).

Echocardiographic characteristics of our patients are described in Table 2 and Table 3.

Regarding LV data, thicknesses, cavity size, as assessed by diameters and volumes, LVM, and LVM/BSA were in the normal range with normal geometry, evaluated by RWT (Table 2). EF and FS were normal, as was CO in the overall population, but a reduction of S wave was observed, suggesting decreased systolic function (Table 2).

With regard to LV diastolic function, the mitral inflow pattern, assessed as E/Apv, showed an impaired relaxation, which was confirmed by a reduction of E′ wave and E′/A′, but E/E′ excluded an increased LV filling pressure in our cohort (Table 2) [18].

Regarding RV, an increase of RVOT was observed, but RVLd and RVTd were in the upper limit of the normal range compared to reference values of ASE (Table 3) [16].

DTE analysis showed RV diastolic dysfunction assessed as a reduction of E′ with an inversion of E′/A′, while S wave was in the lower range. PAP, estimated as AT, was in the lower cutoff value, suggesting an increase of pulmonary resistances in our population, while SPAP was normal (Table 3).

The multivariate analysis showed that the factors independently associated with RV dimensions and PAP, assessed as SPAP and AT, were HRCT score and HI. Both had a positive correlation with RV dimensions and SPAP and a negative correlation with AT. An inverse relationship was also observed between DVT and SPAS but not with AT (Table 4).

With respect to RV diastolic function, the powerful determinants, besides age, were CPAP and chest pain, which negatively influenced some parameters, such as E′tvi and E′/A′tvi (CPAP, E’tvi: *p* < 0.021; E′/A′tvi: *p* < 0.035; chest pain, E′tvi: *p* < 0.018; E′/A′tvi: *p* < 0.02) (Table 4). A significant inverse correlation was also observed between CRP and E/Atvi (*p* = 0.018) and between HI and trans-tricuspidal E/Apv (*p* < 0.05), suggesting that CRP and HI could negatively impact RV diastolic function (Table 4).

RV E/E′ was positively related to D-dimer (*p* < 0.01) and DVT (*p* < 0.02), thus demonstrating the marked activation of coagulation pathways, expressed by higher D-dimer, and this thrombotic complication can influence the central venous pressure even after several months (Table 4). RV systolic function, assessed as S wave at the lateral tricuspidal annulus, was not influenced by COVID-19 disease (data not shown).

With regard to LV, the main determinant of diastolic dysfunction, apart from age, was CRP, which negatively influenced many conventional and tissue diastolic parameters, such as transmitral E/A ratio, septal and lateral E′/A′ ratio, and E′ wave, suggesting that the higher the CRP, the worse the diastolic dysfunction and that there was no tendency towards an increase in E/E′, an index of pulmonary venous pressure (Table 5).

Negative determinants of LV diastolic function were also HI, dyspnea, and thoracic pain (Table 5). Unlike RV, CPAP and hospitalization had a positive effect on LV diastolic function.

With respect to LV, the systolic function, assessed as EF and FS, was not negatively influenced by COVID-19 disease (Table 5).

## 4. Discussion

Most patients who have had COVID-19 are still symptomatic after many months post infection, but the long-term outcomes are not well defined. Few studies have been published on this topic, but active research is ongoing [19,20,21,22,23]. The aim of our research was to define the lasting cardiac impact of this infection.

Our prospective/retrospective study started in May 2020, during the first wave of the pandemic and is part of a larger multidisciplinary project aimed at evaluating the aftermath of COVID-19 in various areas. We studied 160 consecutive patients who were referred for a follow-up visit at University-Hospital of Parma between May and November 2020 and who had the inclusion criteria reported in the Materials and Methods section.

We found that, after an average follow-up of 5 months, most patients still exhibit morpho-functional alterations that involve RV and LV, identified by RV dilation, increased pressure in the pulmonary circulation, and bi-ventricular systolic-diastolic dysfunction. Such changes are significantly correlated with several markers of COVID-19 infection, be they clinical, bio-humoral, or HRCT score.

The determinants of the cardiac changes linked to COVID-19 are different in RV and LV, with RV being more susceptible to HRCT score, and HI and LV being more susceptible to a hyper-inflammation state, suggesting different pathogenic mechanisms involved in the two ventricles.

In the retrospective part of the study, the most frequent symptoms were dyspnea and fever, followed by anosmia and ageusia. Among inpatients, 15% were in a critical state, assisted by mechanical ventilation in ICUs, and had a median hospitalization of 20 days. The prevalence of thrombotic complications, such as VTE or DVT, in inpatients was 13%, followed by arrhythmia (10%), and HI (7%).

Clinical evaluations at follow-up showed that the majority of post-COVID-19 patients still exhibited one or more symptoms, particularly dyspnea, chest pain, and asthenia. Few patients (*n* = 10) reported *restitutio ad integrum*, i.e., the absence of symptoms.

Echocardiography revealed LV diastolic dysfunction, and LV systolic function, as assessed by EF, was conserved, but the DTE showed reductions of S waves.

RV exhibited structural modifications such as dilations of the efflux chamber, and the indices of diastolic function (conventional and DTE) showed deterioration of the relaxation phase. PAP, appraised in all patients by AT, suggested increased vascular pulmonary resistance, whereas a direct estimation of SPAP, which we could only perform in 60% of cases due to the absence of a tricuspid jet, reported values that were within the normal range.

A multivariate analysis (Table 4) revealed that the dimensional RV parameters correlated with two features of the disease, i.e., HRCT and HI; these relationships were independent of age, sex, BMI, SBP, smoking habits, and previous respiratory diseases.

HRCT also correlated with the parameters of pulmonary pressure, i.e., SPAP and AT, suggesting a pathogenic action of pulmonary damage on these variables. Indeed, COVID-19 can trigger an increase of PAP consequent to several alterations, such as parenchymal damage, hypoxic vasoconstrictions, or macro- and micro-thrombosis. We hypothesized that the structural and functional adaptations we observed were due to a pressure overcharge of the RV, subsequent to pulmonary hypertension. Of note, these adaptations were still evident after 5 months, possibly because of the incomplete resolution of parenchymal and vascular pulmonary damage. These data are relevant because our cohort included non-hospitalized and, hence, less severe cases.

Another predictor of both RV dimensions and PAP was the cardiac complication HI. The multivariate analysis used HI as an independent variable, given that our aim was to assess a cause–effect relationship, that is to say, cardiac modifications due to the acute phase of the disease. Yet, we could not exclude reverse causation, in other words, that RV dysfunction caused this complication. In summary, this study demonstrates that the relationship between RV alterations and SARS-CoV-2 infection is one of causality. This hypothesis was corroborated by cardiac imaging studies performed during the acute phase, which showed a principal involvement of the RV, thus revealing prognostic implications [13,14,15,24,25].

Our study confirmed and further extended previous results. In the first systematic echocardiographic examination of 100 consecutive patients requiring hospitalization due to COVID-19 infection, the most common finding was RV dilatation with or without dysfunction (39%), followed by LV diastolic dysfunction (16%) and LV systolic dysfunction (10%) [15]. A second echo was performed in 20% of these cases due to a clinical deterioration during hospitalization, thus showing further degradation of RV parameters (dilatation and dysfunction) associated with shortened AT [15]. The authors hypothesized that the RV changes were secondary to the increase in vascular resistance, evidenced by the shortening of the AT, whose causes were attributable to multiple factors, such as lung parenchymal damage, hypoxic vasoconstriction, pulmonary embolism, or excessive positive end-expiratory pressure [15].

In an echocardiographic study of critically ill patients hospitalized for COVID-19, RV dilatation was associated with higher HRCT score, lower blood pressure, and increased use of vasopressor, thus suggesting a relationship between RV remodeling and lung parenchymal damage and hemodynamic instability, in agreement with our data [25].

Similarly, Li et al. showed that RVLS was a powerful predictor of death in patients with COVID-19, suggesting that an assessment of RV function should be implemented to identify patients at higher risk for poor outcomes [13].

A later published study showed that pulmonary hypertension (with enlarged RV dimensions) in patients with COVID-19 admitted to non-intensive care units was associated with more severe disease and a greater risk of death or transfer to intensive care as compared to patients without pulmonary hypertension [26].

We, in agreement with a previous report [27], did not find significant correlation between the two biomarkers, D-dimer and CRP, or RV structural dimensions or pulmonary pressure parameters. There are some plausible explanations for this lack of correlation, e.g., the period between infection and PAP estimation and the specificity of the latter, which is also a marker of elevated inflammation.

However, D-dimer and DVT both showed a significant positive correlation with RV E/E′, suggesting that high D-dimer levels in the acute phase correspond with worse RV diastolic function and higher right atrial pressure at follow-up. In contrast, CRP showed a negative influence on RV diastolic function, assessed as RV E/A. DTE indices of RV diastolic function, E′ and E′/A′, were inversely related with CPAP and thoracic pain. In contrast, CPAP and Hospitalization positively correlated with DTE-indices of LV diastolic function. It is well known that CPAP may deteriorate RV diastolic function and improve LV diastolic function [28,29]. We speculate that a more intensive treatment in the acute phase, represented by CPAP and hospitalization, made it possible to preserve the LV diastolic function and that this effect could persist even after months, as we observed in the present study.

Based on our results, COVID-19 appears to have effects on LV that are different than those on RV. For example, HRCT score and D-dimer did not influence its function. Conversely, CRP negatively affected all diastolic LV parameters, regardless of age. The association between CRP and LV diastolic function suggests that the acute phase hyper-inflammatory status induced systemic phlogosis, which involved the myocardium. Such phlogosis is probably not due to a direct infiltration of the virus in myocytes, but, rather, is non-specifically due to the increase of IL-6 and TNF-alpha, as previously suggested [4,5,8,9,11,12]. In support of this hypothesis, there is no current evidence of a viral presence in the myocardium, as shown by endomyocardic biopsies of patients with cardiogenic shock or acute myocarditis [9,11,12]. A diffuse macrophage infiltration was reported by Basso et al. in autopsies of 21 patients who died from COVID-19 [30]. Hence, myocardial damage is, conceivably, a consequence of systemic inflammation and a “cytokines’ storm” with possible consequent myocardial fibrosis.

Interestingly, the analysis of systolic function revealed quite normal EF values, which were <55% in only 4% of our cohort (the lower value was recorded in a patient with post-infarction cardiomyopathy), whereas the DTE analysis showed a reduction of S waves in both the Sept. and Lat. walls, significantly influenced by age. On the other hand, CRP was mildly and non-significantly associated with Lat S wave.

We acknowledge as limitations the monocentric and retrospective nature of the present study along with some missing data for the outpatients. In addition, we could not record any data regarding the cardiac clinical condition of our patients prior to SARS-CoV-2 infection or during the acute phase of COVID-19. It should be noted that the sample of enrolled patients cannot be considered as representative of the general population. However, our sample was representative of those subjects who are considered at risk for Long-COVID syndrome based on our local medical classification system, and, therefore, our findings may be pertinent and useful for managing patients in the weeks and months following acute COVID-19 infection. In addition, more sophisticated techniques, such as GLS, were not applied.

Despite these limitations, our study has important strengths. The main one is the wide spectrum of clinical features of the COVID-19 patients under study due to the range of severity from mild to severe cases, thus reflecting the heterogeneity of the SARS-CoV-2 infection. What is more, the echocardiographic examinations were performed by a single, experienced operator to limit the variability of the echocardiographic measures.

## 5. Conclusions

In conclusion, our study confirms cardiac sequelae post-COVID-19, i.e., at 5 months from remission. Notably, echocardiography provides evidence of cardiac changes that involve both ventricles and that are correlated with infection markers independent of age, SBP, sex, and co-morbidities. Infection markers impact RV and LV differently, the former being influenced by HRCT and HI, and the latter by CRP. We speculate that RV suffers from hemodynamic pulmonary alterations in the acute phase, whereas LV is affected by the inflammatory status. Most symptoms, such as dyspnea, still persistent some months after infection and hospitalization, might be the consequence of cardiac and pulmonary distress. This factor certainly deserves further investigation. A long-term follow-up is warranted to refine and calibrate the most appropriate therapeutic strategies.

## Figures and Tables

**Table 1:** Clinical data in the COVID-19 population (*n* = 160).

Baseline Characteristics	*n* (%)
Total	160 (100)
Age (years)	60 ± 12
Male (*n*,%)	96 (60)
BMI (kg/m^2^)	28 ± 5.6
HR (bpm)	73 ± 12.6
SBP (mmHg)	130 ± 16.0
DBP (mmHg)	83 ± 9.6
Smoking (*n*,%)	12 (8)
Former smokers (*n*,%)	68 (43)
Hypertension (*n*,%)	68 (43)
DM (*n*,%)	23 (14)
CAD (*n*,%)	17 (11)
CRDs (*n*,%)	33 (21)
Beta-blockers (*n*,%)	37 (23)
Ace-inhibitors (*n*,%)	29 (18)
ARB (*n*,%)	22 (14)
ASA (*n*,%)	29 (18)

Legend: Data are expressed as mean ± standard deviation or number of subjects with corresponding percentage. ARB, angiotensin receptor blocker; BMI, body mass index; CAD, coronary artery disease; CRDs, chronic respiratory diseases; DBP, diastolic blood pressure; DM, diabetes mellitus; HR, heart rate; SBP, systolic blood pressure.

**Table 2:** Echo-based left ventricular structural and functional data in the total population (*n* = 160).

Variable	Mean ± SD	*n* (%) Abnormal According to Guidelines
End-diastolic diameter (mm)	48.4 ± 4.7	
End-systolic diameter (mm)	29.4 ± 4.5	
Septal wall thickness (mm)	9.4 ± 1.9	
Posterior wall thickness (mm)	8.9 ± 1.4	
End-diastolic volume (mL)	103 ± 27	
End-systolic volume (mL)	34 ± 14	
LVM (g)	195 ± 59	
LVM/BSA (g/m^2^)	102 ± 26	53 (34.2)
Relative wall thickness	0.38 ± 0.06	
Ejection fraction (%)	68 ± 7	
Fractional shortening (%)	39 ± 6	
Cardiac output (mL)	69 ± 16	
Mitral Epv (cm/s)	55.3 ± 12.7	112 (74.7)
Mitral Etvi (cm)	9.2 ± 2.3	
Mitral Apv (cm/s)	68.0 ± 14.2	
Mitral Atvi (cm)	7.4 ± 1.8	
Mitral E/Apv (cm/s)	0.8 ± 0.2	
Mitral E/Atvi (cm)	1.3 ± 0.4	
Sept. Spv (cm/s)	7.4 ± 1.4	
Sept. Stvi	1.5 ± 0.3	
Lat Spv (cm/s)	8.8 ± 2.3	
Lat Stvi (cm)	1.6 ± 0.3	
Sept. E′pv (cm/s)	7.0 ± 2.2	76 (51)
Sept E′tvi (cm)	0.8 ± 0.3	
Lat. E′pv (cm/s	9.4 ± 3.5	93 (61.2)
Lat. E′tvi (cm)	0.9 (0.6–1.1)	
Sept E′/A′pv	0.70 ± 0.26	
Sept E′/A′tvi	0.96 ± 0.35	
Lat. E′/A′pv	0.89 ± 0.45	
Lat. E′/A′tvi	1.1 (0.8–1.6)	
E/E′	6.5 ± 2.9	3 (2)

Legend: Data are expressed as mean ± standard deviation or median (IQ range). BSA, body surface area; Lat, lateral site of mitral annulus; LVM, left ventricular mass; pv, peak velocity; tvi, time velocity integral; Sept., septal site of mitral annulus. (E’ in the E/E’ are the mean of Sept and Lat. Site).

**Table 3:** Echo-based right ventricular structural and functional data in the total population (*n* = 160).

Variable	Mean ± SD	*n* (%) Abnormal According to Guidelines
RVTd (mm)	37.2 ± 7.3	33 (22.6)
RVLd (mm)	66.4 ± 7.6	13 (8.1)
RVOT (mm)	31.7 ± 4.7	30 (19.2)
SPAP (mmHg)	28.0 ± 5.1	9 (8)
AT (ms)	110.9 ± 25.1	68 (44.4)
Tricuspidal Epv (cm/s)	41.2 ± 7.9	
Tricuspidal Etvi (cm)	7.6 ± 1.9	
Tricuspidal Apv (cm/s)	39.1 ± 11.9	
Tricuspidal Atvi (cm)	5.2 ± 2.0	
Tricuspidal E/Apv (cm/s)	1.1 ± 0.3	26 (17.6)
Tricuspidal E/Atvi (cm)	1.6 ± 0.7	
Spv (cm/s)	13.0 ± 2.4	5 (3.3)
Stvi (cm)	2.4 ± 0.4	
E′pv (cm/s)	10.2 ± 2.6	26 (17.1)
E′tvi (cm)	1.5 ± 0.4	
E′/A′pv	0.7 ± 0.2	29 (19.1)
E′/A′tvi	1.1 ± 0.4	
E/E′	4.2 ± 1.5	17 (11.3)

Legend: Data are expressed as mean ± standard deviation. AT, acceleration time; pv, peak velocity; RVLd, RV longitudinal dimension at the end-diastole; RVOT, right ventricular outflow tract; RVTd, RV transversal dimension at the end-diastole; SPAP, systolic pulmonary artery pressure; tvi, time velocity integral.

**Table 4:** COVID-19 alterations during the infection independently related to right ventricle and pulmonary artery pressure at the follow-up: backward regression analysis.

	Output	Predictors Related to COVID-19	β ± SE	*p* Value
RV Dimensions	RVTd	HRCT	0.07 ± 0.03	=0.032
		HI	4.8 ± 2.8	=0.08
	RVLd	HRCT	0.087 ± 0.36	=0.017
		HI	7.96 ± 3.20	=0.015
	RVOT	/	/	/
Pulmonary artery pressure	SPAP	HRCT	0.06 ± 0.027	=0.026
		HI	4.90 ± 2.11	=0.024
		DVT	−4.77 ± 1.96	=0.018
	AT	HRCT	−0.28 ± 0.11	=0.014
		HI	−25.85 ± 10	=0.012
RV Diastolic Function	E/A_pv_	HI	−0.309 ± 0.150	=0.042
		D-Dimer	2.586 ± 0.00	=0.042
	E/A_tvi_	CRP	−0.002 ± 0.001	=0.018
	E′_pv_	/	/	/
	E′_tvi_	CPAP	−0.282 ± 0.119	=0.021
		Thoracic pain	−0.217 ± 0.090	=0.018
	E′/A′_pv_	/	/	/
	E′/A′_tvi_	CPAP	−0.178 ± 0.083	=0.035
		Thoracic pain	−0.166 ± 0.069	=0.019
	E/E′	DVT	1.520 ± 0.644	=0.021
		D-Dimer	000 ± 0.00	=0.011

Legend: The significance of the single factors was weighed for all the other predictors having *p* < 0.1 in the multivariate model, after the stepwise selection method. The full starting model included all the variables mentioned in the statistical analysis paragraph. Abbreviations: CRP, C-reactive protein; DVT, deep vein thrombosis; HRCT, High-Resolution computed tomography; HI, hemodynamic instability; RV, right ventricle.

**Table 5:** COVID-19 alterations during the infection independently related to left ventricle at the follow-up: backward regression analysis.

	Output	Predictors Related to COVID-19	β ± SE	*p* Value
	LVM/BSA	Palpitation	12.2 ± 5.9	=0.043
		CRP	0.061 ± 0.032	=0.063
LV Diastolic function	E/A_pv_	CRP	−0.001 ± 0.00	=0.028
	E/A_tvi_	CRP	−0.001 ± 0.00	=0.004
		HI	−0.309 ± 0.150	=0.039
	E′_pv_ Sept.	CRP	−0.006 ± 0.002	=0.022
		Hospitalization	2.721 ± 0.994	=0.008
	E′_tvi_ Sept.	CRP	−0.001 ± 0.00	=0.025
		Thoracic pain	−0.130 ± 0.054	=0.018
	E′_pv_ Lat.	Hospitalization	3.425 ± 1.431	=0.019
	E′ _tvi_ Lat	CRP	−0.004 ± 0.002	=0.012
		CPAP	1.248 ± 0.326	=0.000
	E′/A′_pv_ Sept.	CRP	−0.001 ± 0.000	=0.003
		Dyspnea	−0.126 ± 0.060	=0.040
		D-Dimer	−0.126 ± 0.060	=0.093
	E′/A′_tvi_ Septal	CRP	−0.001 ± 0.000	=0.018
		Dyspnea	−0.2333 ± 0.092	=0.013
	E′/A′_pv_ Lat.	DVT	−0.301 ± 0.158	=0.061
	E′/A′_tvi_ Lat.	CRP	−0.006 ± 0.002	=0.006
		CPAP	1.605 ± 0.411	=0.000
	E/E′	CRP	−0.007 ± 0.004	=0.043
		Hospitalization	−5.338 ± 1.415	=0.000
		HI	3.123 ± 1.301	=0.019
LV systolic function	EF	/	/	/
	FS	/	/	/
	S_pv_ Sept.	Asthenia	0.557 ± 0.311	=0.078
	S _tvi_ Sept.	HI	0.429 ± 0.136	=0.002
	S _pv_ Lat.	CRP	−0.006 ± 0.004	=0.074
		HRCT	0.029 ± 0.013	=0.030
		DVT	−1.790 ± 0.916	=0.054
	S_tvi_ Lat.	/	/	/

Legend: The significance of the single factors was weighed for all the other predictors having *p* < 0.1 in the multivariate model, after the stepwise selection method. The full starting model included all the variables mentioned in the statistical analysis paragraph. Abbreviations: CRP, C-reactive protein; DVT, deep vein thrombosis; HRCT, High-Resolution computed tomography; HI, hemodynamic instability; LV, left ventricle.

## Supplementary Materials

The following are available online at www.mdpi.com/article/10.3390/diagnostics11112059/s1, Supplementary results. Table S1: COVID-19 severity in hospitalized patients. Table S2: Laboratory data. Table S3: COVID-19 Therapy. Table S4: ECG findings in the total COVID-19 population.

## References

[1] Indolfi C., Spaccarotella C. (2020). The Outbreak of COVID-19 in Italy: Fighting the Pandemic. JACC Case Rep..

[2] Ruan Q., Yang K., Wang W., Jiang L., Song J. (2020). Clinical predictors of mortality due to COVID-19 based on an analysis of data of 150 patients from Wuhan, China. Intensive Care Med..

[3] Shi S., Qin M., Cai Y., Liu T., Shen B., Yang F., Cao S., Liu X., Xiang Y., Zhao Q. (2020). Characteristics and clinical significance of myocardial injury in patients with severe coronavirus disease 2019. Eur. Heart J..

[4] Akhmerov A., Marbán E. (2020). COVID-19 and the Heart. Circ. Res..

[5] Atri D., Siddiqi H.K., Lang J.P., Nauffal V., Morrow D.A., Bohula E.A. (2020). COVID-19 for the Cardiologist: Basic Virology, Epidemiology, Cardiac Manifestations, and Potential Therapeutic Strategies. JACC Basic Transl. Sci..

[6] Inciardi R.M., Lupi L., Zaccone G., Italia L., Raffo M., Tomasoni D., Cani D.S., Cerini M., Farina D., Gavazzi E. (2020). Cardiac Involvement in a Patient with Coronavirus Disease 2019 (COVID-19). JAMA Cardiol..

[7] Bangalore S., Sharma A., Slotwiner A., Yatskar L., Harari R., Shah B., Ibrahim H., Friedman G.H., Thompson C., Alviar C.L. (2020). ST-Segment Elevation in Patients with COVID-19—A Case Series. N. Engl. J. Med..

[8] Guzik T.J., Mohiddin S.A., Dimarco A., Patel V., Savvatis K., Marelli-Berg F.M., Madhur M.S., Tomaszewski M., Maffia P., D’Acquisto F. (2020). COVID-19 and the cardiovascular system: Implications for risk assessment, diagnosis, and treatment options. Cardiovasc. Res..

[9] Imazio M., Klingel K., Kindermann I., Brucato A., De Rosa F.G., Adler Y., De Ferrari G.M. (2020). COVID-19 pandemic and troponin: Indirect myocardial injury, myocardial inflammation or myocarditis?. Heart.

[10] Ruzzenenti G., Maloberti A., Giani V., Biolcati M., Leidi F., Monticelli M., Grasso E., Cartella I., Palazzini M., Garatti L. (2021). COVID and Cardiovascular Diseases: Direct and Indirect Damages and Future Perspective. High Blood Press Cardiovasc. Prev..

[11] Sala S., Peretto G., Gramegna M., Palmisano A., Villatore A., Vignale D., De Cobelli F., Tresoldi M., Cappelletti A.M., Basso C. (2020). Acute myocarditis presenting as a reverse Tako-Tsubo syndrome in a patient with SARS-CoV-2 respiratory infection. Eur. Heart J..

[12] Tavazzi G., Pellegrini C., Maurelli M., Belliato M., Sciutti F., Bottazzi A., Sepe P.A., Resasco T., Camporotondo R., Bruno R. (2020). Myocardial localization of coronavirus in COVID-19 cardiogenic shock. Eur. J. Heart Fail..

[13] Li Y., Li H., Zhu S., Xie Y., Wang B., He L., Zhang D., Zhang Y., Yuan H., Wu C. (2020). Prognostic Value of Right Ventricular Longitudinal Strain in Patients with COVID-19. JACC Cardiovasc. Imaging.

[14] Park J.F., Banerjee S., Umar S. (2020). In the eye of the storm: The right ventricle in COVID-19. Pulm. Circ..

[15] Szekely Y., Lichter Y., Taieb P., Banai A., Hochstadt A., Merdler I., Gal Oz A., Rothschild E., Baruch G., Peri Y. (2020). Spectrum of Cardiac Manifestations in COVID-19: A Systematic Echocardiographic Study. Circulation.

[16] Lang R.M., Badano L.P., Mor-Avi V., Afilalo J., Armstrong A., Ernande L., Flachskampf F.A., Foster E., Goldstein S.A., Kuznetsova T. (2015). Recommendations for cardiac chamber quantification by echocardiography in adults: An update from the American Society of Echocardiography and the European Association of Cardiovascular Imaging. J. Am. Soc. Echocardiogr..

[17] Pelà G., Li Calzi M., Pinelli S., Andreoli R., Sverzellati N., Bertorelli G., Goldoni M., Chetta A. (2016). Left ventricular structure and remodeling in patients with COPD. Int. J. Chronic Obstruct. Pulm. Dis..

[18] Nagueh S.F., Appleton C.P., Gillebert T.C., Marino P.N., Oh J.K., Smiseth O.A., Waggoner A.D., Flachskampf F.A., Pellikka P.A., Evangelista A. (2009). Recommendations for the evaluation of left ventricular diastolic function by echocardiography. J. Am. Soc. Echocardiogr..

[19] Augustin M., Schommers P., Stecher M., Dewald F., Gieselmann L., Gruell H., Horn C., Vanshylla K., Cristanziano V.D., Osebold L. (2021). Post-COVID syndrome in non-hospitalised patients with COVID-19: A longitudinal prospective cohort study. Lancet Reg. Health Eur..

[20] Carfì A., Bernabei R., Landi F. (2020). Persistent Symptoms in Patients after Acute COVID-19. JAMA.

[21] Goërtz Y.M.J., Van Herck M., Delbressine J.M., Vaes A.W., Meys R., Machado F.V.C., Houben-Wilke S., Burtin C., Posthuma R., Franssen F.M.E. (2020). Persistent symptoms 3 months after a SARS-CoV-2 infection: The post-COVID-19 syndrome?. ERJ Open Res..

[22] Halpin S.J., McIvor C., Whyatt G., Adams A., Harvey O., McLean L., Walshaw C., Kemp S., Corrado J., Singh R. (2021). Postdischarge symptoms and rehabilitation needs in survivors of COVID-19 infection: A cross-sectional evaluation. J. Med. Virol..

[23] Stavem K., Ghanima W., Olsen M.K., Gilboe H.M., Einvik G. (2021). Persistent symptoms 1.5–6 months after COVID-19 in non-hospitalised subjects: A population-based cohort study. Thorax.

[24] Baycan O.F., Barman H.A., Atici A., Tatlisu A., Bolen F., Ergen P., Icten S., Gungor B., Caliskan M. (2021). Evaluation of biventricular function in patients with COVID-19 using speckle tracking echocardiography. Int. J. Cardiovasc. Imaging.

[25] Kim J., Volodarskiy A., Sultana R., Pollie M.P., Yum B., Nambiar L., Tafreshi R., Mitlak H.W., RoyChoudhury A., Horn E.M. (2020). Prognostic Utility of Right Ventricular Remodeling over Conventional Risk Stratification in Patients with COVID-19. J. Am. Coll. Cardiol..

[26] Pagnesi M., Baldetti L., Beneduce A., Calvo F., Gramegna M., Pazzanese V., Ingallina G., Napolano A., Finazzi R., Ruggeri A. (2020). Pulmonary hypertension and right ventricular involvement in hospitalised patients with COVID-19. Heart.

[27] Schott J.P., Mertens A.N., Bloomingdale R., O’Connell T.F., Gallagher M.J., Dixon S., Abbas A.E. (2020). Transthoracic echocardiographic findings in patients admitted with SARS-CoV-2 infection. Echocardiography.

[28] MacIntyre N.R. (2019). Physiologic Effects of Noninvasive Ventilation. Respir. Care.

[29] Shim C.Y., Kim D., Park S., Lee C.J., Cho H.J., Ha J.W., Cho Y.J., Hong G.R. (2018). Effects of continuous positive airway pressure therapy on left ventricular diastolic function: A randomised, sham-controlled clinical trial. Eur. Respir. J..

[30] Basso C., Leone O., Rizzo S., De Gaspari M., van der Wal A.C., Aubry M.C., Bois M.C., Lin P.T., Maleszewski J.J., Stone J.R. (2020). Pathological features of COVID-19-associated myocardial injury: A multicentre cardiovascular pathology study. Eur. Heart J..

