# Supplementary material for: Long-Term Cardiac Sequelae in Patients Referred into a Diagnostic Post-COVID-19 Pathway: The Different Impacts on the Right and Left Ventricles
Source: Diagnostics (Basel). 2021 Nov 6;11(11):2059. doi: 10.3390/diagnostics11112059 (PMC8623572; doi:10.3390/diagnostics11112059)
Supplement: Supplementary file 1 [file diagnostics-11-02059-s001.zip › diagnostics-1392080-supplementary.pdf]

Supplementary Results and Tables

Regarding laboratory data, CRP and D-dimer were markedly increased, transaminases slightly increased and creatinine was in the normal range (Table S2).
The pharmacological treatment consisted of Antibiotics (90%), Hydroxycloroquine (72%), Colchicine (14%), Antivirals (lopinavir/ritonavir or darunavir/cobicistat) (49%), Biologics (Tocilizumab or Sarilumab, 10%), Systemic corticosteroids (41%), Low Molecular Weight Heparin (LMWH) in a prophylactic dose (52%), and LMWH in anticoagulant dose (19%) (Table S3).
ECG showed repolarization abnormalities such as ST depression in 24% patients, right intraventricular conduction disturbances in 8%, and 6% showed arrhythmias at follow-up. PR, QRS and QTc intervals were in the normal range (Table S4).

Table S1. COVID-19 severity in hospitalized patients

**Variable**	**Total**<br>**117(73)**
Chest CT visual score	44 ± 21
Length of hospital stay (days)	20.0 (9.0-37.5)
Length of Intensive Care Unit stay (days)	45.0 (37.5-75.5)
Ordinary regime admissions (n,%)	99 (85)
Intensive Care Unit admissions (n,%)	17 (15)
Oxygen therapy (n,%)	104 (89)
Mask (n,%)	69 (66)
Noninvasive ventilation (n,%)	20 (18)
Invasive mechanical ventilation (n,%)	15 (14)
**CV Complications**	
Angina (n,%)	5 (3)
Arryhthmia (n,%)	16 (10)
Myocarditis (n,%)	-
Pericarditis (n,%)	1 (1)
DVT (n,%)	3 (2)
VTE (n,%)	12 (8)
Hemodynamic instability (n,%)	11 (7)

Legend: data are expressed as mean ± standard deviation, median (IQ range) or number of subjects with corresponding percentage. CV, cardiovascular; DVT, deep vein thrombosis; VTE, venous thromboembolism. Chest CT visual score in the total population

Table S2. Laboratory data

Parameters	Total	Normal range
CRP (mg/dL)	104 (37-164) (104)	0.001-5.000
D-dimer (ng/mL)	864 (495-1703) (107)	<500
AST (U/L)	43 (31-65) (108)	0-40
ALT (U/L)	37 (25-53) (109)	0-40
Creatinine (mg/dL)	0.8 (0.7-1.1) (110)	0.5-1.4

Legend. Data are expressed as median (IQ range). In brackets, the number of subjects in which the parameter was available. Alanine transaminase, ALT; AST, aspartate transaminase; CRP, C-reactive protein.

Table S3. COVID-19 Therapy

Variable	Total
Antibiotics (n,%)	144 (90)
Hydroxycloroquine (n,%)	115 (72)
Colchicine (n,%)	22 (14)
Antivirals *(lopinavir/ritonavir or darunavir/cobicistat)* (n,%)	78 (49)
Biologics *(Tocilizumab or Sarilumab)* (n,%)	16 (10)
Systemic corticosteroids (n,%)	65 (41)
LMWH - prophylactic dose (n,%)	83 (52)
LMWH - therapeutic dose (n,%)	30 (19)

Legend. Data are expressed as number of subjects with corresponding percentage

Table S4. ECG findings in total COVID-19 population

Parameters	Total n=160
PR interval (ms)	154 ± 28
Atrial Fibrillation (n)	2% (3/160)
QRS complex duration (ms)	85 ± 14
Right bundle branch block (n)	
• Incomplete	4%(6/160)

• Complete	4%(7/160)
QTc interval (ms)	393 ± 30
ST segment depression (n)	24%(38/160)
Supraventricular premature beats (n)	3%(5/160)
Ventricular premature beats (n)	3%(4/160)

Legend. Data are expressed as mean values ± standard deviation or percentage.
